# Medical Items

**Published:** 1895-10

**Authors:** 


### MEDICAL ITEMS.

PRESS dispatches announce the death of M. Louis Pasteur, September 28th.

---

WE wish the addresses of the following: Drs. J. M. Carswell, W. W. Calhoun, A. Oscar Brown, J. A. Garrett, J. F. Huss, D. B. Harris, J. A. Harper.

The above gentlemen are subscribers who should receive their JOURNALS, but who have failed to furnish us with their new address after leaving college.

We shall be thankful to any one who will furnish the desired information.

---

DR. ROBT. L. VAUGHT, of Chattanooga, while visiting relatives in West Virginia, was drowned in the Kanawha river, August 28th. Dr. Vaught has been for some years Professor of Anatomy in the Chattanooga Medical College, and was highly esteemed both in his social and professional relations.

---

WE have received the Official Report of the First Meeting of the American Academy of Railway Surgeons from the ex-Secretary, Dr. R. Harvey Reed, of Columbus, Ohio. The report contains the Reasons for its organization, its Constitution and By-Laws, and some interesting papers on Railway Surgery. The present Secretary is Dr. Webb J. Kelly, Gallion, Ohio.

---

WE are requested to announce that there will be a special meeting of the Alumni Association of the Southern Medical College in Atlanta, October 18th. Among the exercises will be addresses by Hon. W. C. Glenn, Dr. Thos. S. Powell, and Dr. John R. Shannon of Cabaniss, Ga., the latter representing the Alumni. All graduates of the school are earnestly invited to be present.

FOLLOWING are the newly elected officers of the Mississippi Valley Medical Association: Dr. H. O. Walker, Detroit, president; Dr. Merrill Ricketts, Cincinnati, first vice-president; Dr. F. C. Woodburn, Indianapolis, second vice-president; Dr. H. W. Leob, St. Louis, Mo., secretary; Dr. H. M. Moyer, Chicago, treasurer. The next meeting will be held in St. Paul, October, 1896.

---

IN the late case of Amick *vs.* Reeves, in Chattanooga, the defendant showed that all the parties whom Amick claimed to have "cured," in and around Chattanooga, were dead. Now another one of the dead "cured" is heard from. This time Mr. S. G. Sea, formerly an important employee of the Amick Company. It has not been very long since his circular letter, announcing his remarkable "cure," was scattered broadcast over the country. This concern, we believe, is still being advertised by some reputable journals!

---

A SUIT for damages, which promises to be of interest, and perhaps importance, to doctors, is now pending in St. Louis. Dr. Bernays performed gastrostomy upon a child for stenosis of the esophagus. A photograph of the patient, to show the artificial opening, was made, and several thousand illustrated reprints of the case were sent out by the operator. Also at the operation there were present several physicians and medical students. All of which has highly offended the delicate sensibilities of the child's mother, who now wants $15,000 damages to soothe her lacerated feelings. It is very doubtful if such process will be successful in the courts, but the outcome will be watched with interest.

---

THEY are getting after the "vitapaths" in Cincinnati, and very properly. One of them was lately arrested and fined for practicing without a license. Judge Dustin said, in pronouncing sentence: "Men who knowingly go into a sick room and prevent anything being done for a dying man by silly incantations and laying on of hands are responsible for his death and ought to be on a par with a murderer in the eyes of the law. God help the dying man who relies upon you or any of the so-called graduates of quackery. You speak of vitapathy being of a higher power than medicine, and you say you ordain ministers at the same time you matriculate vitapathic physicians. Your methods are an insult to intelligence, their practice is a criminal abuse of ignorance, and your college a disgrace to civilization." We congratulate the judge upon his elegant characterization.

---

A COMMITTEE has been appointed by the Tri-State Medical Society of Arkansas, Mississippi, and Tennessee, to make a full report on the subject of *Malarial Hematuria* at the next meeting in November. The committee is composed of: Drs. William Krauss, G. G. Buford, and G. B. Malone, of Memphis, and Dr. R. W. Barton, of Marion, Arkansas. Those who have had experience with this disease can assist the committee by giving information on the following points:

1. Number of case. 2. Age, color, sex, etc. 3. Previous history. 4. Sanitary surroundings. 5. Kind of drinking-water. 6. Season of year. 7. Is malaria abundant at the time, and in what year. 8. Is malarial hematuria abundant at the time? 9. What type of malaria, if any, preceded the attack? 10. Had patient taken quinine, and how much? 11. Symptoms, character of urine, etc. 12. Therapy. 13. Was blood examined? 14. Termination. 15. Post-mortem appearance. 16. Is it patient's first attack?

---

THE seventh annual meeting of the Tri-State Medical Society of Alabama, Georgia, and Tennessee promises to be of more than passing interest. The meeting will be held in Chattanooga, Tuesday, Wednesday, and Thursday, October 8th, 9th, 10th. There is every reason to believe that it will be largely attended and of unusual importance. Every doctor in the three States who loves his profession, who desires his own advancement, and who respects ethical medicine should attend this meeting to assist in maintaining the high character of the society, and to foster harmony. If you desire to read a paper, report a case, or present a specimen, please report to the secretary. A cordial invitation is extended to you to attend this meeting to enjoy a delightful recreation, and assist by your presence and your voice in adding to the glories and triumphs of the healing art. R. M. CUNNINGHAM, Pres., Birmingham, Ala.

FRANK TRESTER SMITH, Sec'y, Chattanooga, Tenn.

